# Laparoscopic-Assisted Transgastric ERCP: A Single-Institution Experience

**DOI:** 10.1155/2018/8275965

**Published:** 2018-03-21

**Authors:** Katherine Habenicht Yancey, Lauren Katherine McCormack, Stephen Samuel McNatt, Myron Sheavictor Powell, Adolfo Zachariah Fernandez, Carl Joseph Westcott

**Affiliations:** Department of General Surgery, Wake Forest Baptist Medical Center, Winston-Salem, NC, USA

## Abstract

**Background:**

Laparoscopic-assisted transgastric endoscopic retrograde cholangiopancreatography (LAERCP) is used for treatment in patients after Roux-en-Y gastric bypass (RYGB), where transoral access to the biliary tree is not possible. We describe our technique and experience with this procedure.

**Methods:**

Electronic medical record search was performed from September 2012 to January 2016, identifying patients who underwent LAERCP per operative records. Charts were reviewed for demographic, clinical, and outcomes data.

**Results:**

Sixteen patients were identified. Average time since bypass was 6.9 years, and length of stay was 3.7 days. Five patients underwent simultaneous cholecystectomy. Eleven patients, or 43%, had cholecystectomy more than 2 years previously. ERCP with sphincterotomy was completed in 15 of 16 patients (94%). Our technique involves access to the bypassed stomach via a laparoscopically placed 15 mm port. We observed one major complication of post-ERCP necrotizing pancreatitis. No minor complications nor mortalities were seen in our series.

**Conclusion:**

Biliary obstruction can occur many years after RYGB and cholecystectomy. Our findings suggest that RYGB patients may be at a higher risk of primary CBD stone formation. LAERCP is a reliable option for common bile duct (CBD) clearance; our technique of LAERCP is technically simple and associated with low complication rate, making it appealing to surgeons not trained in advanced laparoscopy.

## 1. Introduction

Roux-en-Y gastric bypass (RYGB) alters gastrointestinal anatomy such that transoral endoscopic retrograde cholangiopancreatography (ERCP) is not routinely feasible. Bariatric surgery patients are predisposed to cholelithiasis, especially within the rapid weight loss period of 12–18 months postoperatively. Cholecystectomy at time of bypass is not routinely performed because ursodiol use postoperatively reduces the incidence of gallstone formation [1, 2]. Nevertheless, patients after gastric bypass will continue to develop indications for ERCP because of choledocholithiasis.

Several approaches exist to access the biliary system in this patient population. Endoscopic intubation of preexisting open gastrostomies was first reported in the 1970s for both gastrointestinal bleeding and ERCP [3]. The first laparoscopic gastrostomy created specifically for ERCP was reported in 2002 [4]. Early techniques for LAERCP included creation of gastrostomy and then allowing several weeks of maturation before use [4, 5]. In 2007, Ceppa et al. [6] described a laparoscopic purse-string technique with immediate intraoperative gastrotomy closure with sutures or staples in ten patients with no surgical complications. Several other techniques have been described; however, they can be technically challenging or require multistep procedures [7–12]. Nonsurgical alternatives with the use of interventional radiology (IR) or endoscopic techniques exist, but they tend to involve external drains and multiple procedures [13–17].

A retrospective review by Brockmeyer et al. [18] studied rates of biliary symptoms after RYGB in 1366 patients. Three hundred and eighty patients had cholecystectomy before RYGB, and three of this group developed primary CBD stones (<1%). Two of the three patients were treated with LAERCP, and one required PTC clearance of the biliary system [17]. These results indicate that primary common duct stone formation was a relatively rare event in this population.

Our aim is to report our technique and experience with a combined endoscopic-surgical procedure for management of biliary obstruction in patients post-RYGB.

## 2. Methods

The study is a retrospective review of operative cases performed at our tertiary referral center in Winston-Salem, North Carolina. After approval by our institutional review board, a chart review of our electronic medical record (EMR) from September 2012 to January 2016 was performed. A keyword search of operative reports was performed using the following terms: ERCP, sphincterotomy, laparoscopic gastrostomy, laparoscopic gastrotomy, laparoscopic cholecystectomy, and cholangiography. Inclusion criteria were age greater than 18, RYGB bariatric surgery patients, and LAERCP dictated in operative report. Indications for ERCP were choledocholithiasis, cholangitis, and radiographic or clinical evidence of common bile duct (CBD) obstruction. Four surgeons and two gastroenterologists were involved. Nineteen patients were identified who met the screening criteria. Of these 19 patients, three patients were excluded because they had CBD clearance laparoscopically. Sixteen patient charts were then reviewed, and information was gathered for analysis, including patient demographics, laboratory and physiologic data, surgical history, operative and endoscopic reports, hospital length of stay, postprocedure outcomes, and follow-up.

## 3. Operative Technique

Our operative technique was similar in all 16 patients; some variability in laparoscopic port placement occurred in those patients undergoing simultaneous cholecystectomy. If recent cholecystectomy had occurred, an open technique was employed to place a periumbilical port through a previous port site. If the abdomen had not been recently accessed, we entered with a 5 mm optical trocar in either the right or left upper quadrant just below the costal margin with the side chosen at the surgeon's discretion. A 12 mm port was placed in the right upper quadrant for passage of the stapler, and it was used if simultaneous cholecystectomy was to be done. Two additional 5 mm ports were placed on either side of the 12 mm port in an arc surrounding the right costal margin. In cases that included cholecystectomy, additional 5 mm ports were placed as per the standard technique. If the patient had a gallbladder present, it was dissected, a cholangiogram was performed, and it was removed via a standard technique. Cholangiogram was performed with intraoperative consult to GI in those patients with RYGB anatomy undergoing cholecystectomy for gallbladder pathology and uncertain preoperative assessment of the common duct. Cholangiograms were not performed in those patients who had previous cholecystectomy as preoperative radiographic assessment was adequate to establish choledocholithasis and need for ERCP.

**Figure 1:**
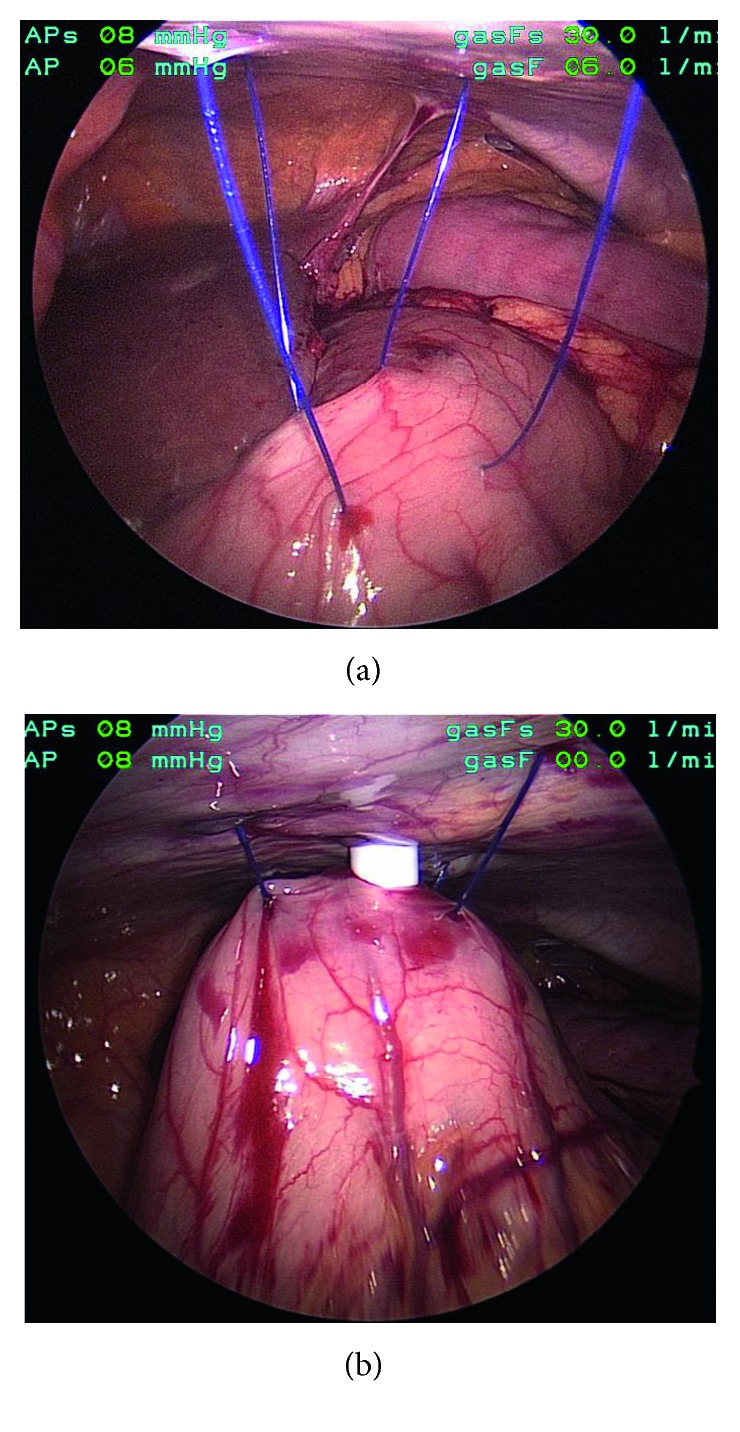
(a) Intraoperative photographs of gastropexy technique. (b) Transabdominal dilation for introduction of 15 mm laparoscopic port for endoscope.

The bypassed stomach was identified, and adhesions were lysed to mobilize the greater curve up to the abdominal wall. An incision was made in the left upper quadrant to accommodate a 15 mm port. Two 2-0 PDS or Prolene sutures were placed through the anterior wall of the stomach on either side of the proposed gastrostomy site. A gastrostomy was then created with cautery. Early in the series, the gastrostomy site was dilated to accommodate a 15 mm port. This step was deemed to be unnecessary, and it was subsequently omitted. A 15 mm port was placed through the abdominal wall and guided through the gastrostomy as stomach was brought to the abdominal wall with the use of the stay sutures (Figure 1). The sutures were then clamped tight to hold the stomach against the abdominal wall. The 15 mm port was then redraped widely for the endoscopy team to proceed with nonsterile ERCP. After the ERCP was completed, the 15 mm port was removed, and the gastrotomy was closed with the use of an endoscopic gastrointestinal stapler using a 3.5 mm staple load. If the patient required repeat endoscopic access of the stomach or biliary tract, the gastrotomy was converted into a Stamm gastrostomy. The patient was either sent home from the postoperative recovery unit or admitted for observation.

## 4. Results

We identified sixteen cases of LAERCP in patients status post-RYGB for morbid obesity. All patients were intraoperatively confirmed to have standard RYGB anatomy. Eleven of sixteen patients (69%) had undergone cholecystectomy before LAERCP. Seven of the eleven, or 43%, had cholecystectomy performed greater than 2 years prior to presentation, with a range of 2–17 years. Four of eleven patients with prior cholecystectomy had cholecystectomy at referring institution, immediately prior to transfer or referral, where intraoperative cholangiogram revealed a filling defect in the CBD. The remaining five patients (31%) underwent simultaneous cholecystectomy with LAERCP at our institution.  Table 1displays our patient's demographic and descriptive data.

The longest time from referral to intervention was seven weeks from the diagnosis of choledocholithiasis. Three patients had LAERCP within 48 hours of direct transfer. Four asymptomatic patients were scheduled electively. Three patients underwent an attempt at transoral ERCP by gastroenterologists at referring institutions prior to referral. The average time between cholecystectomy and LAERCP was nine years (range 0–15 years). Four patients underwent outpatient procedures, defined as hospital stay less than 24 hours. The overall average length of stay was 3.7 days. All patients were initially sent to the floor. One patient who developed post-ERCP pancreatitis required subsequent admission to the intensive care unit.  Table 2 summarizes our perioperative data.

ERCP with sphincterotomy and cannulation of the CBD was successful in 15 of 16 patients (94%). In one patient, the ampulla could not be identified endoscopically. This patient required a percutaneous transhepatic choledochostomy (PTC) drain to relieve CBD obstruction and allow access for duct clearance over a 4-week period. In all fifteen LAERCP procedures, cholangiogram findings were consistent with choledocholithiasis, and balloon-sweep clearance of the duct was successful. In the five cases of simultaneous cholecystectomy, intraoperative cholangiograms via the gallbladder were performed, and all revealed filling defects.

In one patient, a cystic duct stump leak was noted at the time of LAERCP that resulted from the laparoscopic cholecystectomy performed one week priorly. Primary closure of the cystic duct stump could not be performed. A biliary stent was placed via ERCP, and the gastrostomy was converted to a gastrostomy tube for retrieval of the stent after leak resolution at 6 weeks.

One patient was converted to laparotomy with continued endoscopy to locate the ampulla of Vater, which was successful. This patient had a history of laparoscopic RYGB 13 years before and a gastrojejunostomy revision 6 years before LAERCP. A portion of the gastric remnant was resected at laparotomy due to nonviability from extensive adhesiolysis and dissection. The patient subsequently suffered post-ERCP necrotizing pancreatitis with associated critical illness that eventually necessitated video-assisted retroperitoneal debridement (VARD). This was the major morbidity for our series, and no minor complications occurred. All patients underwent successful CBD clearance. There were no mortalities at 30 days.

## 5. Discussion

Our institution's experience illustrates several points not already described in the literature. We describe a technique that can be a single-stage procedure that is technically uncomplicated. Our technique proved to be a useful tool in treating the combination of choledocholithasis and associated cystic duct stump leak. This particular patient had the gastrostomy tube removed 6 weeks after a repeat ERCP and stent removal after confirming resolution of the cystic stump leak. The management of this combination with LAERCP has not been described in the literature before.

We performed five simultaneous cholecystectomy and LAERCP procedures, with four patients undergoing cholecystectomy within 6 weeks of LAERCP. Seven of sixteen patients had remote cholecystectomy with average time before LAERCP of 9 years. For the series, the average time since RYGB was 6.9 years. Interestingly, 43% of our patients presented two or more years after initial cholecystectomy. Our findings suggest that CBD obstruction can occur many years after both cholecystectomy and gastric bypass. This suggests that RYGB may predispose patients to primary CBD stone formation. More investigation into the causes and preventive measures should be considered based on these results.

Familiarity with LAERCP as a means of biliary system clearance for obstructing stones is recommended for surgeons caring for patients who have had RYGB, as this patient population can develop biliary obstruction from stones even after cholecystectomy. The need for LAERCP will only increase as the number of RYGB patients continues to grow, necessitating nonbariatric surgeons to be familiar with the procedure. The technique presented here for access is simple and effective and can be utilized by surgeons without advanced laparoscopic or bariatric surgery training.

We had one failure of the procedure necessitating IR PTC placement with eventual clearance of stones over a 4-week period. We had one complication of post-ERCP necrotizing pancreatitis. Our failure and complication rates are consistent with those reported in the literature [4, 5, 10]. Limitations of our study include its retrospective nature and the small size of the case series. Long-term follow-up was limited, as most of the patients returned to their referring physicians and institutions.

## 6. Conclusion

A subset of patients will develop CBD obstruction after RYGB and require clearance alternatives to standard transoral ERCP. We choose LAERCP because it allowed for the possibility of treating patients with a single-stage procedure, avoiding drain or gastrostomy tube maintenance. The patient population that will require LAERCP is small, but it will increase as RYGB is employed as a treatment for the epidemic of obesity in this country. Primary common bile duct stone formation may be higher than in the general population, as 43% of our patients were two or more years from cholecystectomy at presentation. It is possible that RYGB anatomy and physiology create a predisposition for primary common duct stones. This finding is not well described and needs further research.

## Figures and Tables

**Table 1:** Summary of the patient's descriptive data.

Patient's data (*n*=16)	Mean value	Range
Body mass index (kg/m^2^)	35.1 (±7.39)	(20–50)
Age (years)	55.8	(29–67)
Time since RYGB (years)	6.9	(1–14)
White blood cell count (×10^3^)^∗^	8.4	(3.5–15.3)
Alkaline phosphatase (U/L)^∗^	258.6	(103–441)
Total bilirubin (mg/dL)^∗^	1.6	(0.3–5.1)
Length of stay (days)	3.69	(0–12)
Follow-up (months)	6.82	(0–22)

RYGB = Roux-en-Y gastric bypass; ^∗^laboratory values were only available for 15 of 16 patients.

**Table 2:** Summary of perioperative and operative data.

Operative data	Number of patients (*n*=16)	% of study population
Cholecystectomy ≥2 years priorly	7	43
Cholecystectomy with positive IOC	5	31
Simultaneous cholecystectomy at LAERCP	5	31
Direct hospital transfer for evaluation	10	62
Electively scheduled outpatient cases	8	50
Conversion to open procedure	1	7.6
Complications related to gastric access	0	0
ERCP with ampullary cannulation	15	94
